# Visual Health in Autism Spectrum Disorder: Screening Outcomes, Clinical Associations, and Service Gaps

**DOI:** 10.3390/medicina61101779

**Published:** 2025-10-01

**Authors:** Emine Tınkır Kayıtmazbatır, Hasan Ali Güler, Şule Acar Duyan, Ayşe Bozkurt Oflaz, Banu Bozkurt

**Affiliations:** 1 Department of Ophthalmology, Faculty of Medicine, Selcuk University, 42250 Konya, Türkiye; dr.sulenuracar@gmail.com (Ş.A.D.); draysebozkurtoflaz@gmail.com (A.B.O.); drbanubozkurt@yahoo.com (B.B.); 2 Department of Child and Adolescent Psychiatry, Faculty of Medicine, Selcuk University, 42250 Konya, Türkiye; dr.hasanaliguler@gmail.com

**Keywords:** autism spectrum disorder, visual health, refractive errors, strabismus, stereopsis, screening, testability, underserved care, pediatric ophthalmology

## Abstract

*Background and Objectives*: Children with autism spectrum disorder (ASD) often experience visual problems, yet their ophthalmic health remains underexplored due to testability challenges and limited-service access. This study evaluated ophthalmic screening outcomes in children with ASD and examined whether autism severity influenced ocular findings or cooperation during examinations. *Materials and Methods*: This cross-sectional study included 210 children with ASD (mean age 8.18 ± 4.99 years; 83.3% male). Examinations were conducted in an autism education center using non-contact methods: stereopsis (LANG I stereotest; LANG-STEREOTEST AG, Küsnacht, Switzerland), cover–uncover, and Hirschberg tests for strabismus, Spot Vision Screener (Welch Allyn Inc., Skaneateles Falls, NY, USA) for refractive errors, and Brückner test for red reflex. Autism severity was assessed with the Turkish version of the Adapted Autism Behavior Checklist (AABC). *Results*: Refractive errors were identified in 22.3% of participants: astigmatism in 15.2%, myopia in 5.2% (including 3 high myopia), and hyperopia in 1.9%. Strabismus was present in 11.9%, most commonly intermittent exotropia. Nearly half (49.5%) could not complete stereopsis testing, and a weak positive correlation was observed between AABC scores and the higher absolute spherical equivalent (SE) value between the two eyes (r = 0.173, *p* = 0.044). Children unable to complete stereopsis testing had significantly higher AABC scores (22.66 ± 9.69 vs. 13.39 ± 9.41, *p* < 0.001). Notably, 50 children (23.8%) had never undergone an eye examination prior to this study. *Conclusions*: Ophthalmic findings, particularly astigmatism and strabismus, are common in children with ASD. Greater autism severity was associated with reduced testability and modestly worse refractive error status. These findings suggest that tailored, accessible eye-care approaches and systematic vision screening may help to reduce overlooked visual problems and support more equitable care for children with ASD.

## 1. Introduction

Autism spectrum disorder (ASD) is a complex neurodevelopmental condition, typically presenting with social communication deficits and repetitive behaviors, and influenced by strong genetic as well as other etiological factors [1]. According to U.S. data from 2022, the prevalence of autism spectrum disorder (ASD) among 8-year-old children was reported as 32.2 per 1000 (approximately one in every 31 children). This prevalence varied across states and was found to be about 3.4 times higher in boys (49.2 per 1000) than in girls (14.3 per 1000). Significant differences in prevalence were also observed among racial and ethnic groups [2]. Beyond its core neurobehavioral features, ASD is associated with a broad range of medical comorbidities, among which ocular and visual abnormalities are relatively common [3]. This indicates that visual problems are not incidental but may represent a consistent comorbidity in ASD. In fact, children with congenital visual impairment or decreased visual acuity have long been reported to show an increased risk of ASD, although whether visual impairment is causative or merely correlative remains controversial [3]. This ambiguity underscores the complex bidirectional relationship between ASD and visual function, suggesting that visual abnormalities may not only accompany but potentially influence the neurodevelopmental trajectory of affected children.

Visual function is particularly important in early childhood development, as it contributes to learning, communication, and social interaction, all of which are domains already challenged in children with ASD [4]. This suggests that even subtle disturbances in visual function may disproportionately affect developmental outcomes in this population. Notably, abnormal visual orientation, exploration, and visuospatial perception are consistently observed in ASD and are considered central to early social and cognitive difficulties; however, the extent to which these perceptual deficits parallel autism severity remains unclear. Studies on visual functioning in individuals with ASD indicate a higher prevalence of astigmatism and strabismus, amblyopia, atypical eye movements, and increased susceptibility to subtle visuomotor deficits compared to their neurotypical peers [5,6,7,8]. However, despite consistent reports of elevated ocular morbidity, these studies vary considerably in design and diagnostic criteria, and most did not systematically examine whether the frequency or severity of such findings correlates with autism severity, thereby limiting the generalizability of current evidence. Additionally, evidence suggests that the retinal structure and function in these children may be affected [9]. These biological alterations support the notion that visual abnormalities in ASD extend beyond behavioral manifestations and may involve structural mechanisms. Previous research has shown that ophthalmologic manifestations vary among different ASD subtypes [10]. However, whether the severity of ASD symptoms is associated with greater visual impairment remains unclear, as this relationship has not been systematically explored. As a result, many children with ASD and coexisting visual abnormalities remain undiagnosed or untreated often due to challenges such as limited cooperation during examinations, difficulties in accessing specialized care, and the absence of standardized screening protocols designed for this population [11]. Moreover, communication challenges faced by individuals with ASD frequently hinder cooperation during eye examinations and decrease testability, leading to an underestimation of ocular morbidity.

Recent innovations, such as Social Story–based communication modules, have demonstrated a significant improvement in testability during ophthalmic assessments [12]. Children with ASD often require healthcare services more frequently than their typically developing peers; however, they face several barriers in accessing care, highlighting the necessity for healthcare environments and protocols that are specifically designed to meet their unique needs [13]. These challenges are particularly pronounced in ophthalmology, where issues such as limited cooperation during examinations and the absence of specialized screening protocols contribute to underdiagnosis and undertreatment of ocular conditions. Children with ASD represent an underserved population in ophthalmology, facing disparities in both access to eye care and the timely diagnosis of treatable ocular conditions.

Visual health is a fundamental aspect of overall well-being in children and plays a critical role in their physical, cognitive, social, and emotional development. In educational settings, optimal vision has been linked to higher levels of academic achievement [14]. Routine vision screenings in childhood are crucial for the early detection and management of refractive errors and amblyogenic risk factors [15]. Large cohort studies have shown that preschool screenings are associated with a lower prevalence of amblyopia and better visual outcomes after treatment compared to children who did not undergo screening [16]. However, the feasibility and effectiveness of these screenings in children with ASD remain largely unexamined. Additionally, there is limited understanding of how the severity of autistic symptoms affects ocular health outcomes and the capacity to participate in standardized ophthalmic assessments. Given that more pronounced behavioral symptoms may reduce cooperation during clinical evaluations, exploring the relationship between the severity of ASD and ophthalmic findings yield important insights for clinicians and policymakers.

The present study aims to describe the ophthalmic screening outcomes in children with ASD and to determine whether the severity of autistic symptoms influences ocular findings and the feasibility of screening. While ocular abnormalities have been frequently reported in ASD, the extent to which these findings are associated with autism severity and the practicality of screening approaches has not been systematically explored.

By directly addressing this gap and employing non-touch methods suitable for children with limited cooperation, the present study seeks to provide evidence that may facilitate the development of tailored eye-care strategies, assist clinicians in anticipating potential examination challenges, and ultimately help to reduce disparities in ophthalmic health among children with ASD.

## 2. Materials and Methods

This study received approval from the Local Ethics Committee (decision no: 2024/524). Informed consent was obtained from the parents of all participants, and the research was conducted in accordance with the principles outlined in the Declaration of Helsinki. All ophthalmic examinations were performed at the Selçuklu Foundation for the Education of Individuals with Autism (SOBE). Children with a confirmed clinical diagnosis of autism spectrum disorder (ASD) who were eligible for routine ophthalmic screenings were included.

All examinations were conducted by a single pediatric ophthalmologist (ETK), with the support of trained educators from SOBE. The standardized protocol included stereopsis assessment with the LANG Stereotest I (LANG-STEREOTEST AG, Küsnacht, Switzerland), evaluation of fixation and eye movements, strabismus assessment by cover–uncover and Hirschberg tests, measurement of refractive errors with the Welch Allyn Spot Vision Screener, and red reflex examination with the Brückner test.

Age-appropriate reference values for refractive errors were applied according to the manufacturer’s instructions for the Welch Allyn Spot Vision Screener (version 3.0.xx.yy; Welch Allyn Inc., Skaneateles Falls, NY, USA) and were strictly followed during test administration and interpretation (Table 1).

In brief, hyperopia, myopia, astigmatism, and anisometropia were categorized based on diopteric cut-off values validated for pediatric populations. These thresholds have been shown to provide acceptable sensitivity and specificity for detecting amblyopia risk factors when compared with cycloplegic refraction [17]. To minimize discomfort and potential risks in this special population, only non-contact methods were employed. Cycloplegic eye drops were not administered, as the examinations were conducted outside the hospital environment and aimed to ensure feasibility in children with limited cooperation. While we acknowledge that non-cycloplegic refraction may underestimate hyperopia due to accommodation, its use is supported by validation studies demonstrating adequate accuracy in community-based screening contexts [17]. This approach was therefore considered appropriate for the primary aim of non-invasive screening, while recognizing cycloplegic refraction as the gold standard for precise measurement. For interocular comparisons, analysis was based on the higher absolute spherical equivalent (SE) value between the two eyes. This definition was strictly based on SE; cylinder and axis parameters were not considered. Appointments for detailed examinations have been scheduled at the hospital for children diagnosed with refractive errors, strabismus and other pathologies.

Autism severity was evaluated using the Turkish version of the Adapted Autism Behavior Checklist (AABC) [18]. The scores obtained were used to explore potential correlations between ophthalmic findings and autism severity.

### Statistical Analysis

All analyses were conducted using SPSS Statistics version 25. Demographic and clinical data were summarized using descriptive statistics. Continuous variables were presented as mean ± standard deviation (SD), and categorical variables as frequencies and percentages. The normality of distribution for continuous variables was evaluated using skewness and kurtosis values, with values between −2 and +2 considered indicative of an approximately normal distribution [19] or the Shapiro–Wilk test, whichever criterion supported normality. Variables fulfilling the normality assumption were analyzed with parametric tests (independent-samples *t*-test, Pearson correlation), whereas those not meeting the assumption were analyzed with non-parametric tests (Spearman correlation). Based on this assumption, comparisons of autism severity (AABC scores) according to stereopsis examination status (exam performed vs. not performed) were carried out using the independent-samples *t*-test.

Correlation analyses were conducted to examine the associations between autism severity and ophthalmic parameters. Pearson correlation analysis was applied for axis variables (right and left eye axis), while Spearman correlation analysis was used for all other refractive parameters (sphere, cylinder, and SE). Additionally, to evaluate the association between autism severity and the higher absolute SE while adjusting for potential covariates (age, sex, and stereopsis examination status), partial correlation analyses were performed. Because this variable did not conform to a normal distribution, a rank transformation was applied before calculating the partial correlation coefficients.

To account for potential confounding factors, a multivariate logistic regression analysis was conducted with stereopsis testability (exam performed vs. not performed) as the dependent variable, and age, sex, AABC score, presence of strabismus, presence of refractive error, and the higher absolute SE value as independent predictors. Odds ratios (OR) with 95% confidence intervals (CI) were reported. A *p*-value < 0.05 was considered statistically significant in all analyses.

## 3. Results

A total of 210 children with ASD participated in the study, with 35 females (16.7%) and 175 males (83.3%). The mean age of the participants was 8.18 ± 4.99 years. Parental reports indicated that 50 children (23.8%) had never undergone an ophthalmologic examination prior to this study. Additionally, 44 children (21.0%) had previously been examined by an eye specialist, while in 116 cases (55.2%), parents were uncertain about the children’s previous eye-care history.

Strabismus was detected in 25 participants (11.9%), of whom 16 (7.6%) had intermittent exotropia, 8 (3.8%) had esotropia, and 1 (0.5%) had congenital sixth nerve palsy. Additionally, 5 children exhibited pseudoesotropia, and 2 had a history of strabismus surgery. Stereopsis assessment could not be performed in 104 participants (49.5%) due to limited cooperation. Of the children who were assessed, stereopsis was absent in 4 cases (1.9%). Stereopsis was measured at 1200 arcsec in 5 participants (2.3%), and at 550 arcsec in 97 participants (46.1%). In the analyses of ASD severity, the mean AABC score was 18.16 ± 10.48 (Table 2).

The refractive status assessment revealed that 163 participants (77.6%) had no significant refractive error. Myopia was detected in 11 children (5.2%), of whom 3 had high myopia (>6 diopters). Hyperopia was found in 4 participants (1.9%), while 32 children (15.2%) exhibited astigmatism. Additionally, anisometropia was identified in 5 children. Regarding laterality, SE values showed no interocular difference in 54 children (25.7%). The mean eye axis was 86.05 ± 74.65° in oculus dexter (OD), and 91.68 ± 71.37° in oculus sinister (OS). The mean spherical value was 0.50 ± 1.31 D OD and 0.38 ± 1.30 D OS. The mean cylindrical value was −0.85 ± 0.80 D OD and −0.75 ± 0.80 D OS. The mean SE was 0.77 ± 1.22 D OD, 0.11 ± 1.21 D OS, and 0.07 ± 1.31 D in the eye with the higher absolute SE. The mean values of refractive and visual parameters are summarized in Table 3.

**Figure 1:**
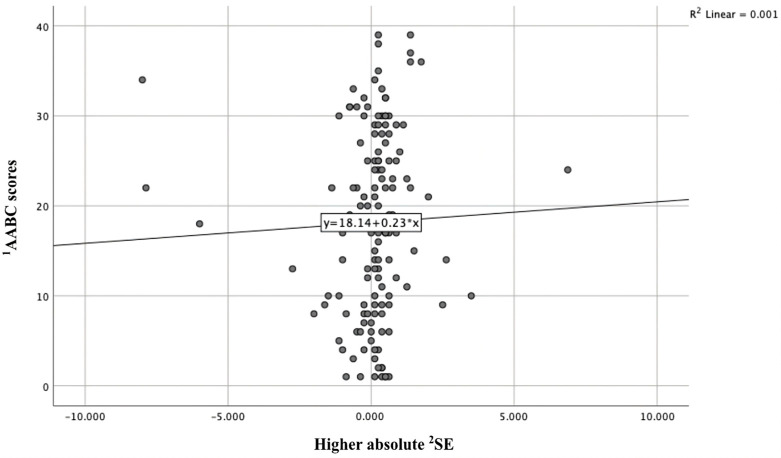
Scatterplot illustrating the correlation between the spherical equivalent (SE) of the eye with higher SE and Adapted Autism Behavior Checklist (AABC) scores. The regression line indicates a weak but statistically significant positive association (r = 0.173, *p* = 0.044). ^1^ AABC: Adapted Autism Behavior Checklist. ^2^ SE: Spherical Equivalent.

Among the remaining participants, the eye with the higher absolute SE was OS in 72 children (34.3%) and OD in 84 children (40.0%). Preliminary analysis indicated a weak positive correlation between the higher absolute SE value and ASD severity scores (r = 0.173, *p* = 0.044; Table 4, Figure 1). Partial correlation analysis, controlling for age, sex, and stereopsis examination status, revealed a modest but statistically significant association between AABC score and the eye with the higher absolute SE value (partial r = 0.176, *p* = 0.042).

Other significant findings included one case of unilateral ptosis, which contributed to anisometropia and astigmatism, one case of bilateral posterior polar cataract, and six children already using spectacles. One child had a history of bilateral retinal detachment surgery accompanied by nystagmus, while three were born prematurely, and five were part of twin pairs, including two pairs in which both twins were diagnosed with ASD.

When comparing autism severity based on the status of stereopsis examination (Table 5), children testable for stereopsis assessment had lower AABC scores (13.39 ± 9.14) compared to those who could not be examined (22.66 ± 9.69) (t = 5.724, *p* < 0.001).

A multivariate logistic regression analysis was conducted to identify independent predictors of stereopsis testability. The overall model was statistically significant (χ^2^(6) = 37.78, *p* < 0.001), with acceptable goodness-of-fit (Hosmer–Lemeshow χ^2^(8) = 6.43, *p* = 0.600) and an overall correct classification rate of 72.1%. Among the predictors, both younger age (OR = 1.17, 95% CI = 1.05–1.31, *p* = 0.005) and lower AABC scores (OR = 0.90, 95% CI = 0.87–0.94, *p* < 0.001) were significantly associated with higher likelihood of successful stereopsis examination. Gender, strabismus status, presence of refractive error, and the higher SE were not significantly related to testability (*p* > 0.05) (Table 6). Each one-point increase in AABC scores was associated with a 10% reduction in the likelihood of completing stereopsis testing.

## 4. Discussion

In this study, we examined the ophthalmic screening findings of children with ASD to explore their potential association with autism severity. Refractive errors have been extensively investigated in children with ASD, although the reported prevalence varies according to methodology, population, and refractive thresholds. Scharre and Creedon, for example, identified significant refractive errors in 44% of their participants aged 2–11 years using near retinoscopy, with hyperopia and astigmatism being the most common findings [20]. However, the study sample was drawn from a special education school, which may have introduced selection bias, and non-cycloplegic refraction was employed, likely underestimating hyperopia. Ezegwui et al. [21] similarly reported notably high rates of astigmatism in Nigerian children with ASD. In contrast, Kabataş et al. [22] and others applying the AAPOS spectacle prescribing criteria observed lower overall rates of myopia and hyperopia, reflecting the influence of more conservative thresholds [7,8]. More recent studies employing cycloplegic autorefraction and standardized epidemiological definitions provide a clearer comparison with typically developing peers, such as Anketell et al. [23], demonstrated no significant differences in myopia or hyperopia between ASD and control groups, but a markedly higher prevalence of astigmatism in children with ASD (25.7% vs. 8.4%). Taken together, these findings suggest that astigmatism may be the most consistently elevated refractive error subtype in ASD, whereas hyperopia and myopia rates may approximate those seen in the general population when measured under cycloplegia.

A systematic review estimated the prevalence of refractive errors in children to be 11.7% for myopia, 4.6% for hyperopia, and 14.9% for astigmatism [24]. Although our study did not include a control group, comparisons with such population-based data provide valuable context for interpreting our findings. In our cohort, refractive errors were identified in 22.4% of children, including 15.2% with astigmatism, 5.2% with myopia, and 1.9% with hyperopia. These values are remarkably close to our results, particularly for astigmatism. At the national level, Gürsoy et al. [25], conducted a cycloplegic screening study and reported 22.6% myopia, 10.6% hyperopia, and 11.0% astigmatism among 7–8-year-old schoolchildren. Compared with these findings, our cohort demonstrated lower rates of myopia and hyperopia but a similar prevalence of astigmatism. The urban, school-based design and the inclusion of cycloplegic refraction likely contributed to higher hyperopia detection, while the elevated myopia rates may reflect environmental and ethnic factors specific to that cohort. This discrepancy may also be partly explained by reduced near work and less engagement in reading or other close-range activities among children with ASD. Additionally, the absence of cycloplegic refraction in our study may have underestimated hyperopia. Taken together, these comparisons suggest that the refractive error burden in children with ASD is broadly in line with both global and Turkish population estimates, with astigmatism consistently emerging as the most frequent subtype.

Beyond prevalence estimates, the clinical significance of refractive errors in ASD lies in their potential educational and developmental consequences. Reynolds et al. [26] showed that surgical correction of high refractive errors in children with ASD not only improved visual acuity but also led to better social interactions and behavioral outcomes in the majority of cases. Similarly, a systematic review by Do et al. [27], highlighted that children with visual impairment have a markedly higher risk of ASD compared with the general population, underscoring how reduced sensory input can compound developmental vulnerabilities. These findings support the view that timely detection and correction of refractive errors in ASD may play a crucial role in optimizing learning, social engagement, and overall developmental trajectories.

Importantly, we observed a weak positive correlation between autism severity scores and the eye with higher SE (r = 0.173, *p* = 0.044). Although modest, this finding suggests that visual impairment could contribute to or coexist with greater autism severity. Reduced visual input may exacerbate difficulties in communication and daily functioning, while lower cooperation during refraction in children with more severe ASD could also account for this association. These results underscore the importance of routine vision screening in ASD, as uncorrected refractive errors may further impair learning and social interaction.

In our study, strabismus was observed in 11.9% of the children, with intermittent exotropia being the most prevalent type, followed by esotropia and one instance of congenital sixth nerve palsy. This prevalence is somewhat lower than prior reports, which have indicated rates ranging from 15% to over 40% in ASD populations. For instance, Black et al. [8] reported strabismus in 41% of their patients, revealing a broad range of horizontal and vertical deviations. Similarly, Ikeda et al. [7] reported a prevalence of 21%, primarily featuring accommodative esotropia and exotropia. In contrast, Gutiérrez et al. [10] described strabismus in 15.4% of a large Spanish ASD cohort, with exotropia being the most common type—aligning closely with our findings. While our prevalence is lower than that reported in specialty clinic–based samples, it is higher than the 8.6% prevalence observed in the retrospective cohort study by Kabataş et al. [22]. The variability across studies may underscore the heterogeneity of ocular motor findings in ASD. Notably, we found no correlation between strabismus and autism severity scores, suggesting that ocular misalignments in ASD may be influenced by shared neurodevelopmental mechanisms rather than behavioral severity. Nevertheless, given the potential impact of strabismus on binocular vision, stereopsis, and overall quality of life, early identification and management remain critical.

In this study, we successfully assessed stereopsis using the LANG I stereotest in just over half of the children with ASD (106 out of 210). Among those testable, 46.1% (97 children) demonstrated stereopsis at the 550 arcsec level, which is generally regarded as within the normal range for this method. Only 4 children (1.9%) showed no stereopsis, while 5 (2.3%) had a reduced stereopsis level of 1200 arcsec. These findings suggest that many children with ASD can achieve near-normal stereopsis levels when a sufficient level of cooperation is present.

Although most of the cooperative children in our cohort demonstrated near-normal stereopsis with the LANG I test, previous research has indicated that impaired stereopsis may be associated with reduced motor coordination and, indirectly, social difficulties [28]. This discrepancy highlights the importance of considering both methodological differences in stereopsis assessment and the potential functional impact of subtle binocular vision deficits on daily living and social participation in children with ASD. For instance, Coulter et al. [29] employed a stepwise stereoacuity testing protocol, starting with the Random Dot 2 and moving to simpler tests (Random Dot E and Lang I) if initial attempts failed. They found that while most typically developing children were able to complete the most challenging test, some children with ASD required simpler tests, and exhibited significantly poorer stereoacuity compared to controls. This highlights how test selection and difficulty can strongly influence stereopsis outcomes in ASD populations. LANG I, being non-verbal and less demanding, may yield better results in ASD samples. However, previous studies using stepwise test protocols have also demonstrated that even when simpler stereotests are available, some children with ASD still struggle to complete them. In our cohort, nearly half of the participants could not be assessed for stereopsis due to difficulties with cooperation which may bias interpretations. In addition, multivariate logistic regression analysis indicated that age and lower AABC scores were independently associated with higher stereopsis testability, whereas gender, strabismus, refractive error, and SE were not significant predictors. This finding supports the notion that developmental and behavioral severity, rather than ocular factors, primarily influence cooperation during stereopsis assessment. This is consistent with the significant effect of behavioral severity on cooperation; in our cohort, each one-point increase in AABC scores was associated with a 10% reduction in testability, underscoring the practical challenges of conducting ophthalmic screening in children with more severe ASD.

Gutiérrez et al. [10] found that ophthalmologic manifestations varied among different subtypes of ASD, with the prevalence of strabismus and refractive errors differing across these groups. Unlike their focus on diagnostic categories, our study examined whether autism severity correlates with ocular findings. We found that while ophthalmic abnormalities are common in children with ASD, they do not strongly correlate with autism severity based on standardized behavioral assessments. This suggests that such ophthalmic findings should be viewed as comorbidities rather than severity markers. Future research with larger cohorts and longitudinal designs are warranted to explore whether early identification and correction of visual problems can influence developmental outcomes. Our findings also stress the value of multidisciplinary collaboration among pediatric ophthalmologists, educators, and autism specialists to ensure comprehensive care for this vulnerable population.

Our findings reveal a significant gap in vision care access for children with ASD. More than half of parents (55.2%) could not report prior eye exams, and among those who could, over half of the children had never seen an eye specialist. This is consistent with findings from the Autism Treatment Network (ATN) Registry, where only 57% of school-aged children with ASD had undergone an eye examination by a vision care professional within the past two years [11]. These findings reflect broader evidence of healthcare disparities in ASD, likely driven by challenges in exam cooperation, limited access and continuity of eye care and low caregiver awareness [30,31].

The high proportion of “unknown” responses highlights shortcomings in care continuity and medical record communication. Taken together, these findings emphasize the necessity for structured, ASD-specific screening protocols, routine integration of vision checks into developmental care, and improved caregiver education to ensure timely detection and management of ocular morbidity in this vulnerable population.

Given the difficulties children with ASD and their families often experience when visiting hospitals—such as sensory overload, transportation issues, and long waiting times—it is essential to explore alternative care pathways. A practical strategy could involve implementing basic ophthalmic screenings directly within ASD education and rehabilitation centers, carried out by ophthalmologists in collaboration with trained educators. Conducting simple, non-contact tests in these familiar settings could facilitate effective triage and enable the early identification of children who need further referral for comprehensive eye care. In cases where significant pathology is suspected, hospital-based examinations could be scheduled during dedicated time slots specifically designed to accommodate the needs of this population. Such an approach has the potential to reduce barriers to access, enhance early detection rates, and ultimately promote more equitable eye care delivery for children with ASD.

The present study has several methodological limitations. First, the absence of a control group of typically developing children restricts direct comparison and limits the generalizability of the findings. Second, refractive error assessment was performed without cycloplegia, which may have led to an underestimation of hyperopia due to accommodation. Although validated non-cycloplegic screening thresholds were applied and supported by previous studies, cycloplegic refraction remains the gold standard. Third, potential selection bias cannot be excluded, as participants were recruited from a specialized center and families with higher awareness or access to care may have been overrepresented. Additionally, the high proportion of non-testable children for stereopsis assessment could introduce bias and obscure true binocular function. Despite these limitations, the study has notable strengths, including the use of non-contact, validated screening methods in a non-clinical environment, the standardized application of test protocols by a pediatric ophthalmologist, and the collaboration with trained educators, which enhanced feasibility in this underserved population. Taken together, these strengths highlight the potential for implementing adapted screening protocols in real-world ASD settings, while the limitations should be considered when interpreting the generalizability of our findings.

## 5. Conclusions

This study demonstrates that ocular findings such as refractive errors and strabismus are common in children with ASD, yet many remain undetected due to testability challenges and limited access to care. Nearly one in four children had never undergone an eye examination, underscoring the need for improved screening strategies. Greater autism severity was associated with reduced testability and modestly worse refractive error status, suggesting that behavioral challenges may influence both cooperation and outcomes. Implementing non-contact screening protocols within ASD education centers, supported by multidisciplinary collaboration, may help overcome barriers, ensure earlier detection, and promote more equitable access to vision care for this vulnerable population.

## Figures and Tables

**Table 1:** Manufacturer’s referral criteria for ocular screening using the Welch Allyn Spot Vision Screener. * D = Diopter.

Age (Months)	6–12	12–36	36–72	72–240	240–1200
Anisometropia D *	1.5	1	1	1	1
Astigmatism D *	2.25	2	1.75	1.5	1.5
Myopia D *	2	2	1.25	1	0.75
Hyperopia D *	3.5	3	2.5	2.5	1.5
Anisocoria (mm)	1	1	1	1	1
Gaze Vertical (degrees)	8	8	8	8	8
Gaze Nasal (degrees)	5	5	5	5	5
Gaze Temporal (degrees)	8	8	8	8	8
Gaze Asymmetry (degrees)	8	8	8	8	8

**Table 2:** Sociodemographic and clinical characteristics of the participants. ^1^ SD = Standard Deviation. ^2^ AABC = Adapted Autism Behavior Checklist. ^3^ SE = Spherical Equivalent. ^4^ D: Diopter.

	Participants (*n* = 210)
Age (Mean ± SD ^1^)	8.18 ± 4.99
Sex: *n* (%)	
Female	35 (16.7)
Male	175 (83.3)
AABC ^2^ Score (Mean ± SD ^1^)	18.16 ± 10.48.
Presence of strabismus: *n* (%)	25 (11.9)
Intermittent exotropia	16 (7.6)
Esotropia	8 (3.8)
Congenital 6th nerve palsy	1 (0.5)
Stereopsis assessment: *n* (%)	106 (50.4)
Absent stereopsis	4 (1.9)
1200 arcsec	5 (2.3)
550 arcsec	97 (46.1)
Presence of refractive error: *n* (%)	47 (22.3)
Myopia	11 (5.2)
High myopia (>6 D ^4^)	3 (1.4)
Hyperopia	4 (1.9)
Astigmatism	32 (15.2)
Laterality: *n* (%)	
No interocular difference	54 (25.7)
Higher absolute SE ^3^: Left	72 (34.3)
Higher absolute SE ^3^: Right	84 (40.0)

**Table 3:** Mean values of refractive and visual parameters. ^1^ SD = Standard Deviation. ^2^ SE = Spherical Equivalent. ^3^ D: Diopter.

	Mean ± SD ^1^
Right Eye Axis (°)	86.05 ± 74.65
Left Eye Axis (°)	91.68 ± 71.37
Right Eye Sphere (D ^3^)	0.50 ± 1.31
Left Eye Sphere (D ^3^)	0.38 ± 1.30
Right Eye Cylinder (D ^3^)	−0.85 ± 0.80
Left Eye Cylinder (D ^3^)	−0.75 ± 0.80
Right Eye SE ^2^ (D ^3^)	0.77 ± 1.22
Left Eye SE ^2^ (D ^3^)	0.11 ± 1.21
Higher absolute SE ^2^: (D ^3^)	0.07 ± 1.31

**Table 4:** Pearson and Spearman Correlation Coefficients between AABC Scores and Ophthalmic Parameters. ^1^ AABC: Adapted Autism Behavior Checklist. ^2^ SE: Spherical Equivalent. ^3^ D: Diopter.

		AABC ^1^ Score	Right Eye Axis (°)	Left Eye Axis (°)	Right Eye Sphere (D ^3^)	Right Eye Cylinder (D ^3^)	Left Eye Sphere (D ^3^)	Left Eye Cylinder (D ^3^)	Right Eye SE ^2^ (D ^3^)	Left Eye SE ^2^ (D ^3^)	Higher Absolute SE ^2^ (D ^3^)
AABC score	r	1.00	0.041	0.022	0.063	0.034	0.023	−0.027	0.153	0.089	0.173
	p		0.637 *	0.802 *	0.467	0.692	0.789	0.757	0.075	0.302	0.044

* Pearson correlation analysis was performed.

**Table 5:** Comparison of Autism Severity by Examination Status. ^1^ AABC = Adapted Autism Behavior Checklist.

	Stereopsis Exam: Yes (*n*: 102)	Stereopsis Exam: No (*n* = 108)	t	*p*
AABC ^1^ Score	13.39 ± 9.14	22.66 ± 9.69	5.724	<0.001

**Table 6:** Logistic Regression Analysis of Adapted Autism Behavior Checklist Score. ^1^ CI: Confidence Interval. ^2^ SE: Spherical Equivalent. ^3^ D: Diopter.

Variable	Beta (Coef.)	Standard Error	Wald Z	*p*-Value	Odds Ratio (OR)	95% CI ^1^ Lower	95% CI ^1^ Upper
Constant	0.393	0.625	22.180	0.529	1.482	-	-
Age	0.157	0.056	7.738	**0.005**	1.170	1.047	1.307
Gender	0.073	0.541	0.018	0.893	1.075	0.372	3.108
Presence of Strabismus	−0.208	0.630	0.109	0.741	0.812	0.236	2.791
Presence of Refractive Error	−0.289	0.510	0.321	0.571	0.749	0.276	2.035
Eye with higher SE ^2^ (D ^3^)	0.075	0.140	0.289	0.591	1.078	0.819	1.419

Reference group = Stereopsis not testable. Odds ratio > 1 indicates a higher likelihood of successful stereopsis examination as the predictor increases. The bolded terms indicate significance levels (*p* < 0.05).

## Data Availability

The raw data supporting the conclusions of this article will be made available by the authors on request.

## Abbreviations

The following abbreviations are used in this manuscript:

ASD	Autism Spectrum Disorder
AABC	Adapted Autism Behavior Checklist
CI	Confidence Interval
D	Diopter
OD	Oculus Dexter
OS	Oculus Sinister
OR	Odds Ratio
SE	Spherical Equivalent
SOBE	Selçuklu Foundation for Individuals with Autism Education
SD	Standard Deviation

## References

[1] Lord C., Elsabbagh M., Baird G., Veenstra-Vanderweele J. (2018). Autism spectrum disorder. Lancet.

[2] Shaw K.A., Williams S., Patrick M.E., Valencia-Prado M., Durkin M.S., Howerton E.M., Ladd-Acosta C.M., Pas E.T., Bakian A.V., Bartholomew P. (2025). Prevalence and early identification of autism spectrum disorder among children aged 4 and 8 years—Autism and Developmental Disabilities Monitoring Network, 16 sites, United States, 2022. MMWR Surveill. Summ..

[3] Reynolds M., Culican S.M. (2023). Visual autism. Children.

[4] Zhou R., Xie X., Wang J., Ma B., Hao X. (2023). Why do children with autism spectrum disorder have abnormal visual perception?. Front. Psychiatry.

[5] Chang M.Y., Gandhi N., O’Hara M. (2019). Ophthalmologic disorders and risk factors in children with autism spectrum disorder. J. AAPOS.

[6] Little J.A. (2018). Vision in children with autism spectrum disorder: A critical review. Clin. Exp. Optom..

[7] Ikeda J., Davitt B.V., Ultmann M., Maxim R., Cruz O.A. (2013). Brief report: Incidence of ophthalmologic disorders in children with autism. J. Autism Dev. Disord..

[8] Black K., McCarus C., Collins M.L., Jensen A. (2013). Ocular manifestations of autism in ophthalmology. Strabismus.

[9] Wang J.E., Tsai S.J., Chen T.J., Wang T.J., Chen M.H. (2023). Risk of retinal disease in patients with autism spectrum disorder. CNS Spectr..

[10] Gutiérrez C., Santoni J.L.M., Merino P., de Liaño P.G. (2022). Ophthalmologic manifestations in autism spectrum disorder. Turk. J. Ophthalmol..

[11] Lindly O.J., Chan J., Fenning R.M., Farmer J.G., Neumeyer A.M., Wang P., Swanson M., Parker R.A., Kuhlthau K.A. (2021). Vision care among school-aged children with autism spectrum disorder in North America: Findings from the Autism Treatment Network Registry Call-Back Study. Autism.

[12] Venkatesh S., Shwetha T.S., Janarthanan S.D., Naaz S., Srinivasan K. (2025). Development and Impact of a Communication Module on Eye Examination Testability Among Individuals with Autism Spectrum Disorder. J. Autism Dev. Disord..

[13] Kolukisa T., Cinar N. (2025). Mothers of children with autism spectrum disorder: Their views on their children’s hospital experiences, expectations from nurses, and a hospital environment sensitive to differences—A qualitative study. Arch. Psychiatr. Nurs..

[14] Neitzel A.J., Wolf B., Guo X., Shakarchi A.F., Madden N.A., Repka M.X., Friedman D.S., Collins M.E. (2021). Effect of a randomized interventional school-based vision program on academic performance of students in grades 3 to 7: A cluster randomized clinical trial. JAMA Ophthalmol..

[15] Williams C., Northstone K., Harrad R.A., Sparrow J.M., Harvey I., ALSPAC Study Team (2002). Amblyopia treatment outcomes after screening before or at age 3 years: Follow up from randomised trial. BMJ.

[16] Williams C., Northstone K., Harrad R.A., Sparrow J.M., Harvey I., ALSPAC Study Team (2003). Amblyopia treatment outcomes after preschool screening v school entry screening: Observational data from a prospective cohort study. Br. J. Ophthalmol..

[17] Peterseim M.M., Papa C.E., Wilson M.E., Davidson J.D., Shtessel M., Husain M., Cheeseman E.W., Wolf B.J. (2014). The effectiveness of the Spot Vision Screener in detecting amblyopia risk factors. J. AAPOS.

[18] Özdemir O., Diken I.H. (2019). Reliability and validity studies of the Adapted Autism Behaviour Checklist in Turkey. J. Dev. Phys. Disabil..

[19] George D., Mallery M. (2010). SPSS for Windows Step by Step: A Simple Guide and Reference, 17.0 Update.

[20] Scharre J.E., Creedon M.P. (1992). Assessment of visual function in autistic children. Optom. Vis. Sci..

[21] Ezegwui I.R., Lawrence L., Aghaji A.E., Okoye O.I., Okoye O., Onwasigwe E.N., Ebigbo P.O. (2014). Refractive errors in children with autism in a developing country. Niger. J. Clin. Pract..

[22] Kabataş E.U., Ozer P.A., Ertugrul G.T., Kurtul B.E., Bodur S., Alan B.E. (2015). Initial ophthalmic findings in Turkish children with autism spectrum disorder. J. Autism Dev. Disord..

[23] Anketell P.M., Saunders K.J., Gallagher S., Bailey C., Little J.A. (2016). Profile of refractive errors in European Caucasian children with autistic spectrum disorder: Increased prevalence and magnitude of astigmatism. Ophthalmic Physiol. Opt..

[24] Hashemi H., Fotouhi A., Yekta A., Pakzad R., Ostadimoghaddam H., Khabazkhoob M. (2018). Global and regional estimates of prevalence of refractive errors: Systematic review and meta-analysis. J. Curr. Ophthalmol..

[25] Gursoy H., Basmak H., Yaz Y., Colak E. (2013). Vision screening in children entering school: Eskisehir, Turkey. Ophthalmic Epidemiol..

[26] Reynolds M., Faron N., Hoekel J., Tychsen L. (2024). Refractive surgery to correct visual impairments in 267 children with autism spectrum and related neuro-developmental disorders: Improvements in vision and behavior. Med. Hypothesis Discov. Innov. Ophthalmol..

[27] Do B., Lynch P., Macris E.M., Smyth B., Stavrinakis S., Quinn S., Constable P.A. (2017). Sys-tematic review and meta-analysis of the association of Autism Spectrum Disorder in visually or hea-ring impaired children. Ophthalmic Physiol. Opt. J. Br. Coll. Ophthalmic Opt..

[28] Smith D., Ropar D., Allen H.A. (2018). Does stereopsis account for the link between motor and social skills in adults?. Mol. Autism.

[29] Coulter R.A., Bade A., Jenewein E.C., Tea Y.C., Mitchell G.L. (2021). Near-point findings in children with autism spectrum disorder and in typical peers. Optom. Vis. Sci..

[30] Bishop-Fitzpatrick L., Kind A.J.H. (2017). A scoping review of health disparities in autism spectrum disorder. J. Autism Dev. Disord..

[31] Muskat B., Greenblatt A., Nicholas D.B., Ratnapalan S., Cohen-Silver J., Newton A.S., Craig W.R., Kilmer C., Zwaigenbaum L. (2016). Parent and health care provider perspectives related to disclosure of autism spectrum disorder in pediatric emergency departments. Autism.

